# Therapist-patient correspondence in internet-based CBT for chronic pain: Associations with outcome and adherence

**DOI:** 10.1016/j.invent.2026.100958

**Published:** 2026-06-04

**Authors:** Nils Gasslander, Felicia Sundström, Gerhard Andersson, Monica Buhrman

**Affiliations:** a Department of Psychology, Uppsala University, Uppsala, Sweden; b Department of Behavioural Sciences and Learning, Department of Biomedical and Clinical Sciences, Linköping University, Linköping, Sweden; c Department of Clinical Neuroscience, Karolinska Institutet, Stockholm, Sweden

**Keywords:** Internet-based therapy, CBT, Online communication, Therapist behaviors, Participant behaviors, Adherence

## Abstract

**Background:**

Guided Internet-based Cognitive Behavioral Therapy (iCBT) typically involves text correspondence with a clinician who provides feedback and answers questions during the course of the treatment. Previous research indicates that therapist guidance can improve outcomes. A few studies show that therapists' type of feedback, and possibly participant's choice of questions, are associated with treatment adherence and outcome. While a small number of studies have been published on this topic, few has reported on both therapist and patient correspondence in the same study, and to our knowledge no studies have been published on correspondence in iCBT for patients with chronic pain.

**Aims:**

The aim of the current study was to characterize therapist-participant correspondence and to examine whether specific behaviors correlate with treatment adherence and/or clinical outcomes in a guided, individually tailored iCBT for individuals with chronic pain and psychological distress.

**Methods:**

Data were obtained from a previously published randomized controlled trial (Gasslander et al., 2022). The study included 1240 therapist messages and 609 participant messages, which were coded using two separate coding schemes. Proportions of coded behaviors were analyzed through correlations to explore associations between therapist-participant communication patterns, treatment adherence (defined as the number of completed treatment steps), and treatment outcomes (pre-to post treatment change scores).

**Results:**

Participant and therapist behaviors were identified and comparisons with previous studies revealed notable similarities and differences. Correlational analyses showed associations both between and within therapist and participant behaviors. No significant associations were observed between specific behaviors and outcome. However, several observed behaviors demonstrated significant correlations with treatment adherence.

**Conclusions:**

Distinct therapist and participant behaviors can be identified in guided iCBT for individuals with chronic pain. While the relationship with treatment outcome is unclear, adherence to treatment seems to be related to the specific types of behaviors exhibited in the text-based correspondence.

## Highlights

• Text-based correspondence is essential in internet-based treatments.
• Few studies have investigated correspondence in treatments of chronic pain.
• Content analysis was used to characterize therapeutic correspondence.
• Distinct participant and therapist behaviors were identified.
• Specifics of therapist-participant correspondence might impact adherence.

## 1 Introduction

Internet-based Cognitive Behavioral Therapy (iCBT) has the advantage of bypassing some of the barriers face-to-face treatments encounter such as geography and stigmatization, by allowing equal access to participants no matter their location or circumstances (Hedman et al., 2012). Results of iCBT for several psychiatric and somatic disorders have been found to be equivalent as face-to-face Cognitive Behavioral Therapy (CBT), and especially therapist supported iCBT (Hedman-Lagerlöf et al., 2023). ICBT for chronic pain has been found to be efficacious regarding interference with day-do-day activities, catastrophizing, pain and mood variables (Buhrman et al., 2016; Terpstra et al., 2022). As with traditional CBT, iCBT can be individually tailored to the patient. Such tailoring might be especially relevant for complex patients often presenting with multiple comorbid issues, where a standardized treatment might be insufficient. This tailoring process can be conducted in various ways (Moggia et al., 2024). For example, using diagnostic criteria, questionnaire data, or patient preferences to assign a sequence of treatment modules.

Despite the evidence for efficacy, effect sizes in iCBT studies on pain have been small to moderate and drop-outs is a problem (Buhrman et al., 2016). Previous research has shown that adherence predicts outcome in both CBT (Jensen et al., 2023; Kazantzis et al., 2010), and in iCBT (Donkin et al., 2011; Hadjistavropoulos et al., 2016). Engagement with treatment content is regarded as a fundamental component of change, as it facilitates the acquisition and application of new skills in everyday life (Kazantzis et al., 2018).

Reducing attrition in iCBT could be a way to improve treatment outcomes, both by retaining participant engagement though long treatment periods, as well as potentially reducing the number of participants who drop out early. However, little is known about which factors most effectively prevent attrition from iCBT. While previous research has identified several early predictors of low adherence in iCBT, such as treatment credibility, treatment personalization, and depression (Alaoui et al., 2015; Beatty and Binnion, 2016; Gasslander et al., 2021; Sanabria-Mazo et al., 2025), and some research suggest that therapist guidance can play a role (Baumeister et al., 2014), less is known about which specific types of guidance and therapist-client interactions are most effective.

Guided iCBT typically involves asynchronous text-based communication with a therapist, who provides individualized feedback and responds to participant questions during the course of the treatment (Vlaescu et al., 2016). In contrast, *unguided* iCBT functions more as a pure-self-help treatment where participants navigate the treatment independently, sometimes supported only by automated messages. Previous research indicates that therapist contact can improve outcomes (Karyotaki et al., 2021), and while the evidence is mixed in studies of chronic pain, therapist contact has been shown to moderate pain self-efficacy (Terpstra et al., 2022). A few studies have also reported that characteristics of this communication might indicate who will benefit most from internet delivered treatments, as well as assist treatment providers in improving their communication methodology (Schneider et al., 2016; Svartvatten et al., 2015).

Several previous studies have been published on text-based correspondence in internet-delivered psychological treatments. Paxling et al. (2012) assessed the contents of e-mails sent by therapists to participants (*n* = 44) in iCBT for individuals with Generalized Anxiety Disorder (GAD). Eight distinct behaviors were identified and coded: Alliance Bolstering, Deadline Flexibility, Empathetic Utterance, Psychoeducation, Self-Disclosure, Self-Efficacy Shaping, and Task Reinforcement. Most of the behaviors correlated significantly and positively with each other, with the exception of Deadline Flexibility which did not show any significant correlation with the other therapist behaviors. Several of the therapist behaviors correlated with treatment adherence (i.e., module completion), and therapist Task Reinforcement correlated with reduced worry, while Deadline Flexibility were correlated with increased worry. Another study (Schneider et al., 2016) used Paxling’s (2012) coding scheme (with three added categories; “Questionnaire Feedback”, “Clarifying Questions”, and “Administrative Statements”) to analyze e-mails sent to patients (*n* = 41) in an iCBT for depression. As in the Paxling (2012) study, a majority of the therapist behaviors showed a large number of significant positive correlations with the other therapist behaviors. This study however, also reported correlations between a majority of the therapist behaviors and *worse* treatment outcome, with the strongest correlations being between Prompting and increased depression and anxiety from pre- to post-treatment. Although these results can appear counter, a higher number of therapist messages is likely to be associated with participants showing low adherence or other treatment related difficulties. This is particularly true for prompting messages, which are used to promote adherence.

Schneider et al. (2016) compared frequencies of the identified behaviors compared to the earlier Paxling (2012) study, and found significant differences in the proportions of behaviors displayed by the therapists. In the earlier study (Paxling et al., 2012), Self-Efficacy Shaping was commonly observed (34%), and Alliance Bolstering was rare (6%), while in Schneider's (2016) sample, Self-Efficacy Shaping was rarely observed (3%), while Alliance Bolstering was the most frequently observed of all the therapist behaviors (30%). Finally, another study (Holländare et al., 2016) conducted a similar analysis on therapist behaviors in an iCBT for depression, and found that several of the nine identified behaviors (mainly “Encouraging” and “Affirming” behaviors, corresponding roughly to Alliance Bolstering in the current study) correlated with adherence, while three were related to outcome.

When it comes to participant text correspondence, a few previous studies have analyzed word use, and findings include shifts in what words participants used over time (Dirkse et al., 2015), and relationships between the types of words used and both outcome and adherence (Van der Zanden et al., 2014). Two studies investigated message content more broadly. One study (Svartvatten et al., 2015) analyzed emails in an iCBT for depression (*N* = 29), and found statements relating to the therapeutic alliance or observing positive consequences to be related to outcome, while many behaviors correlated with treatment adherence. Soucy et al. (2019) described the content of patient questions in iCBT for depression and anxiety (*N* = 80). Results showed that questions pertaining to the treatment material were the most frequent (47%), followed by questions regarding the therapeutic process (23%). The study showed no correlation between the types of questions asked and treatment adherence or outcome.

In summary, therapists type and style of feedback can influence treatment credibility and adherence, as well as having a direct impact on participant mood and behaviors (Holländare et al., 2016; Paxling et al., 2012). For participants, the amount and type of questions posed could indicate different levels of treatment credibility, adherence and engagement (Dirkse et al., 2015; Soucy et al., 2019). While a few studies have been published on this topic, most studies focus on therapist behaviors, and to our knowledge, no previous clinical trials have reported both therapist and patient correspondence in the same study (González-Robles et al., 2024). Correspondence in guided iCBT is inherently dyadic however, involving reciprocal communication between therapists and participants. Each individual's behavior can influence the other, shaping the course and quality of the interaction. By systematically describing and analyzing both therapist and participant contributions, it may be possible to uncover patterns that could give new insights on the relational dynamics of therapeutic correspondence underpinning adherence and engagement. Such insights could inform the development of more effective communication strategies aimed at optimizing treatment outcomes. Finally, there are few if any studies published on communication in iCBT for patients with chronic pain.

The aim of the current study was to describe characteristics of therapist and participant communication, as well as to clarify whether communication patterns between therapists and participants relates to adherence and outcome in a guided and individually tailored internet delivered cognitive behavioral therapy (iCBT) for individuals with chronic pain and psychological distress. Given the current limited understanding of factors influencing adherence in iCBT, the results from this study could give new insights into potential predictors of both adherence and outcome.

## 2 Methods

### 2.1 Treatment

All data were collected in a study on individually tailored iCBT for chronic pain and comorbid psychological distress conducted during 2016–2018 (Gasslander et al., 2022). The trial was approved by the Swedish Ethical Review Authority (2016/107). Participants were recruited from a specialist pain clinic, and inclusion criteria required patients to have a chronic pain condition, have been recently assessed by a physician, and report psychological distress. The treatment consisted of 20 modules, of which three were mandatory (containing introductory information, mid-treatment maintenance, and treatment summarization, respectively), while the rest were used for tailoring. Each participant was assigned three to nine additional modules, resulting in a six to eight week-long treatment. Module choice was based on participants initial interviews, and potential themes of the tailored modules were anxiety, communication, depression, insomnia, relaxation, stress, trauma and worry.

A digital treatment platform used in regular care at the Uppsala University Hospital was used to host the treatment and collect data. Participants used two-factor authentication to access the treatment platform using a personal computer, and could then communicate with their assigned therapists using a messaging service on the secure platform. For more details on the treatment, see the original study (Gasslander et al., 2022).

### 2.2 Therapists and participants

Study participants were 70% female and around 45 years old on average with a long history of chronic pain (mean of 15 years). All reported some form of phycological distress, and had previous contact with specialist care. The current sample includes seventy-seven of the original 95 participants (81%) who used the option to message their therapists. The dataset included 609 messages sent by the participants, with each participant sending an average of 6.4 messages (SD = 6.7) during the treatment. See Table 1. For additional details on participant demographics, see the original study (Gasslander et al., 2022).

**Table 1:** Therapists, participants, and messages.

Therapists (n)	9
Age (mean [SD])	25.5 [2.3]
Female (n [%])	8 [88.8]


Participants (n)	95
Age (mean [SD])	45.6 [11.1]
Female (n [%])	70 [73.7]
Pain duration, years (mean [SD])	15.4 [11.1]
Pre-treatment Depression (mean [SD])^a^	20.5 [9.5]
Pre-treatment Pain Interference (mean [SD]^)^^b^	4.4 [1.1]
Treatment adherence (mean % [SD])^c^	50.4 [37.3]
Working/studying full time (n [%])	26 [27.4]


Messages (n)	1849
Messages sent to participants (n, mean, range [SD])	1240, 6.4, 0–31 [6.7]
Messages received by participants (n, mean, range [SD])	609, 13.1, 1–36 [6.93]

a Measured by the Montgomery-Åsberg Depression Rating Scale (MADRS).

b Measured by the Multidimensional Pain Inventory (MPI).

c Proportion of completed/assigned modules.

Therapists were eight students at the end of their five-year clinical psychologists' education (MSc-level), and one licensed psychologist (NG). The therapists were between 24 and 31 years old and one had previous experience working with iCBT for chronic pain in specialist care. All nine had previous training and clinical experience in CBT. The therapists received both preparatory training and weekly supervision from an experienced psychologist, psychotherapist and researcher in chronic pain (MB). Before the treatment started, all therapist were instructed in the use of relevant software, received guidance on how to adapt their previous training to the internet-based format, and as well as a detailed walkthrough of the treatment content. For correspondence, therapists were instructed to focus on alliance building and adherence facilitation by communicating interest in the participants experiences, using active listening skills and empathetic statements, explaining difficult treatment concepts, prompting treatment related behaviors and assisting in practical (often IT-related) issues with the treatment platform or content. Therapists were also instructed to avoid giving advice or instructions outside the scope of the treatment. Therapists sent a total of 1240 messages to participants, with each therapist sending an average of 137.8 total messages, and an average of 13.1 messages per participant (SD = 6.9) during the treatment. See Table 1.

### 2.3 Measures

All measurement data were exported from the internet-based platform, where the outcome measures were the two primary outcome questionnaires (MADRS and MPI – Pain Interference) completed by the participants. No data were missing at pretreatment, but 31% of posttreatment data were missing.

MADRS. The self-report version of the Montgomery-Åsberg Depression Ration Scale (MADRS-S) was one of the two main outcomes in the original study. MADRS-S is a 9-item questionnaire measuring depressive symptoms on a scale from 0 to 6, resulting in a total score of 0–54 (Svanborg and Åsberg, 1994).

Pain Interference. The Multidimensional Pain Inventory (MPI) measures consequences of chronic pain and the Swedish version includes five subscales. The subscale Pain Interference was the second main outcome of the original study, and measures the degree to which pain interferes with daily functioning using 11 items on a scale from 0 to 6, resulting in a total score of 0–66 (Bergström et al., 1998).

Adherence data was based on the number of modules where participants had completed all mandatory assignments. Two measures were used; (1) the proportion of completed modules, calculated as the number of completed modules divided by the total number assigned (range: 0–1), and (2) a binary indicator of treatment completion, defined as having completed at least 75% of the assigned modules (coded as 1 for completion and 0 for non-completion).

### 2.4 Coding

Before the coding procedure, text correspondence from the therapists was extracted and anonymized by removing all identifying information from the texts. All messages were then coded according to two different coding schemes (Table 2 and Table 3). Coding was based on directed qualitative content analysis (Hsieh and Shannon, 2005), where the data were first explored using previously identified coding schemes as a basis, and recurring themes observed that did not fit a code where assigned new categories. This preliminary coding was first carried out using the schemes developed by Paxling et al. (2012) for therapist behaviors, and Soucy et al. (2019) for participant behaviors. The results of the preliminary exploration and observed deviations from the original schemes were then discussed with the other investigators, after which final coding schemes were developed. For participant messages, the final scheme consisted of five behaviors: four question categories (Facilitate Understanding, Therapy Process, Technical Issues, and Information Requests), and a “Non-question” category. For therapist messages, the final scheme consisted of nine behavior categories (Deadline Flexibility, Task Reinforcement, Alliance Bolstering, Prompting, Psychoeducation, Self-Disclosure, Self-Efficacy Shaping, Empathetic Statements and Administrative Statements). For more details on these schemes, see Table 2, Table 3.

**Table 2:** Coding categories for participant messages.

Behavior category	Specification	Example
Facilitate Understanding	Questions on undertaking or applying the treatment content	“I don't quite understand the exercise about values. Could you explain?”
Therapy Process	Questions on the treatment process, goals or procedures.	“Will you send feedback on the exercises I completed?
Technical Issues	Practical or technical questions on how to use the treatment platform.	“I can't find the button to complete the module, could you help me?”
Information Request	Requests for information or resources outside the treatment context.	“Could you recommend a good physiotherapist?”
Non-questions	Statements not requiring or expecting a specific reply. Such as self-disclosing, rhetorical questions, or informing the therapist of treatment progress.	“I've had a good week and finally managed to complete week 2!”

**Table 3:** Coding categories for therapist messages.

Behavior category	Specification	Examples
Deadline Flexibility	Expressing lenience regarding deadlines.	“It's no problem if you send your exercises next week instead.”
Task Reinforcement	Reinforcement of (specific) completed tasks in the treatment, such as homework exercises.	“You have clearly understood the exercise and identified several important behaviors, well done!”
Alliance Bolstering	Showing an interest for participants situation. Specific questions, advice and suggestions.	“I'm curious to know more about… “ “My suggestion would be to take a break between…”
Prompting	Asking the participant to complete a task or expressing an interest in a specific result.	“How's it going with the final exercise?”
Psychoeducation	Providing information on psychological processes, theory, goals of treatment, etc.	“The purpose of this exercise is…” “A SMART goal can make it easier to tell…”
Self-Disclosure	Mentioning personal experiences.	“I have also struggled with depression and…”
Self-Efficacy Shaping	Prompting or reinforcing participants engaging in health behaviors outside treatment exercises.	“Great work with changing the way you approach your job; it seems you're really applying what you've learnt”
Empathetic Statements	Conveying understanding or empathy for a specific difficulty.	“That sounds terrible, I'm sorry you have had such a hard time sleeping”
Administrative Statements	Providing technical or practical information.	“To access next week's material, you'll need to click the button that says…”

The initial exploration highlighted an issue with following Paxling's (2012) scheme, since it relied on data being segmented into paragraphs. Since no paragraphs existed in the present study's data, all messages were instead divided into *functional statements*, where repeated statements pertaining to the same specific topic was only coded once, even when they occurred disconnected from each other in the message. For example, a participant might initiate their message by asking for help with printing a worksheet, move on to a different question, and later in the message get back to the topic of printing. These two parts of the message (related to printing) would then be counted as part of the same functional statement, while a different IT-related question in the same message (e.g., log-in problems) would be counted as a separate statement. In summary, statements in any given coding category *could* be coded several times in one message, though repeated statements pertaining to the same topic were not coded again. Additionally, only one code per statement was allowed, and for statements potentially fitting several categories, an assessment of the primary function of the statement was made in order to select the most appropriate category. This assessment was based on clinical judgement and contextual clues from the conversations being coded. For example, the statement “I'm curious to see what you thought about this week's assignment” could be construed as fitting both Prompting and Alliance Bolstering categories. By assessing message history, coders might note that the participant was late in sending in their homework, and as such determined that the statement was primarily Prompting in nature.

#### 2.4.1 Participant correspondence

For participant correspondence, directed qualitative content analysis (Hsieh and Shannon, 2005) was carried out using the coding scheme developed by Soucy (2019) as a starting point. The focus on participant questions were chosen since instructions to the participants focused on using the messaging function to ask questions of their therapists. Data was all text messages from participants (*n* = 77) to their therapists (*n* = 9). All types of questions defined by Soucy were observed in the preliminary exploration, except “Information Request”, indicating participants in this sample did not ask their therapists for information or resources outside the treatment. Nevertheless, the category was retained for the main coding, in order to enable a comparison with the previous study. Preliminary analysis also indicated that while questions were frequent in participant messages, many messages contained no questions. The scheme focused on participant questions, coding all questions into one of Soucy's (2019) categories, while statements that weren't questions were coded as a fifth behavior category (Non-questions). See Table 2 for participant coding scheme.

Coding of participant behaviors was carried out by a licensed clinical psychologist and PhD-student (NG) and followed by a separate licensed psychologist and PhD-student (FS) co-coding a randomly selected 15 messages. Inter-rater reliability was assessed by correlational analyses between coders for these 15 messages, and revealed significant correlations for all observed behaviors. Facilitate Understanding had perfect correspondence (*r* = 1.0), and none of the coders observed any Information Requests. For Technical Issues and Non-questions, correlations were strong to very strong (*r* = 0.78/0.88, *p* < 0.01), though for the category Therapy Process a moderate correlation was obtained (*r* = 0.54, *p* < 0.05).

#### 2.4.2 Therapist correspondence

Data included all text communication from the therapists in the study (n = 9) to their assigned participants (*n* = 95). A directed qualitative content analysis approach was used. The coding scheme used as a basis was developed by Paxling et al. (2012). All behaviors defined by the original scheme were observed in the preliminary exploration, except “Self-Disclosure”, indicating therapists in this sample did not volunteer information on their personal experiences when messaging their participants. The category was retained for the main coding, in order to enable comparisons with previous studies. Finally, one additional category (“Administrative Statements”) were identified as a recurring theme, and was added to the final scheme, following a later study (Schneider et al., 2016). The final scheme divides statements into one of 9 behavior categories. See Table 3 for therapist coding scheme.

Coding of therapist behaviors was carried out by two licensed clinical psychologists and PhD-students (NG and FS). Initial coding was conducted in tandem, with repeated discussions and refinement of the coding schema in order to reach consensus. Subsequent coding was carried out separately, with only infrequent discussions on difficult cases. Coding was followed by co-coding of randomly selected 10% (*n* = 125) of messages. Randomization was conducted using an online random number generator (random.org), and was stratified to three thirds of the data, based on time, in order to not over-represent early messages. Inter-rater reliability was assessed by correlational analyses between coders for these 125 messages, and revealed significant correlations (*p* < 0.01) between coders for all observed behaviors. No Self-Disclosure or Self-Efficacy Shaping was observed by any of the coders in the double-coded sample. Very strong correlations (*r* > 0.8) were observed between coders in the categories Task Reinforcement, Psychoeducation and Non-questions. Strong correlations (*r* > 0.6) between coders were observed for Deadline Flexibility, Alliance Bolstering and Empathetic Statements. Finally, Prompting correlated moderately between coders (*r* = 0.57).

### 2.5 Analysis

All statistics were carried out using SPSS version 28. Descriptive statistics were calculated to describe the total, range, and average of coded messages for each behavior category. Independent *t*-tests were used to investigate whether frequencies of coded behaviors differed between participants with clinically significant, or clinically non-significant outcomes. Note that significance in this context is determined by the relationship between the difference in group means, the variability within groups, and the sample size. Consequently, a small absolute difference may reach statistical significance if within-group variability is low, and variables with larger absolute differences may not be statistically significant if there is greater variability or wider dispersion within the groups.

To avoid potential issues with analyzing behavior frequencies given the high individual variability in treatment lengths and the amount of correspondence, relative *proportions* of coded behaviors were computed for each participant. Correlational analyses were then based on these proportions (behaviors relative to the total amount of behaviors coded for each participant/therapist). However, an analysis based on behavior frequencies are included in Supplement A. Correlations were computed to assess relationships between the coded behaviors, and between coded behaviors and measures of both adherence (completed modules) and outcome. Spearman's rho was used due to the non-parametric nature of the data.

For outcome, residualized change scores on the MADRS and MPI-Pain Interference scales were calculated by converting to z-values, and subtracting pre- from posttreatment scores, after multiplying the pretreatment scores with the residuals (Z_post_ - Z_pre ⁎_ r_pre-post_). A positive change score thus denotes improvement, while a negative score signifies deterioration.

## 3 Results

### 3.1 Behaviors and intercorrelations

**Fig. 1:**
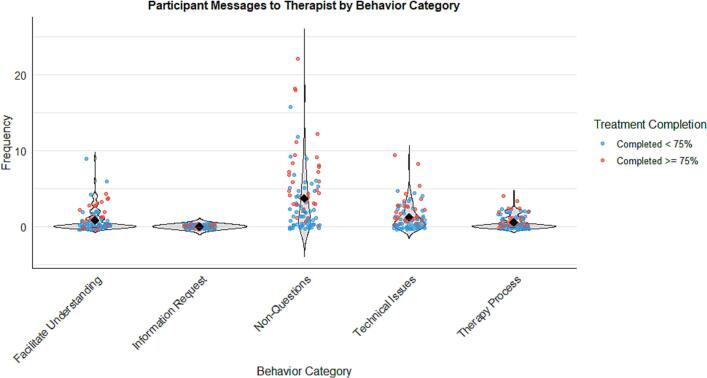
Frequencies of participant to therapist messages per behavior category. Colors show non-completers (blue) and completers (red).

In total 596 participant behaviors were coded, with the most frequently observed being non-questions (59%) followed by Technical Issues (20%), Facilitate Understanding (13%), and Therapy Process (9%). Requests for information outside the treatment was never observed in this dataset (0%). See Fig. 1 for frequencies of all participant behaviors.

**Fig. 2:**
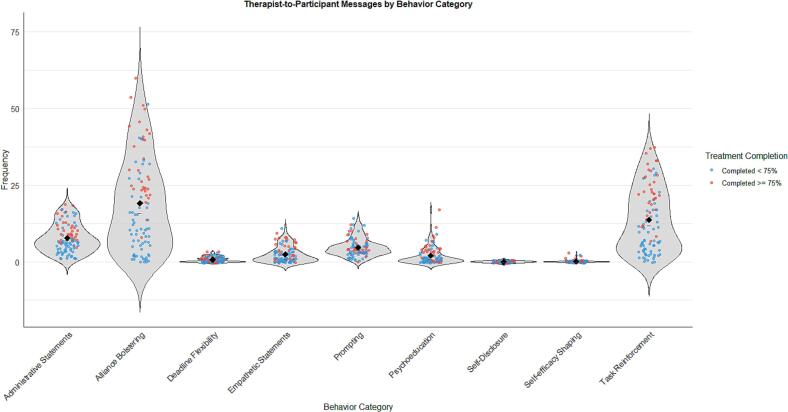
Frequencies of therapist to participant messages by behavior category. Colors show non-completers (blue) and completers (red).

For therapists, 4788 behaviors were coded, the most common one being Alliance Bolstering (38%), followed by Task Reinforcement (27%) and Administrative Statements (15%). No Self-Disclosure by therapists were observed in this dataset (0%). See Fig. 2 for frequencies of all therapist behaviors.

Participants who attained clinically significant change (defined as ≥30% improvement from pre- to post-treatment) were compared to those improved <30%, using independent *t*-tests. Results showed that participants who reached clinical significance on the MADRS sent significantly more Non-questions (*t* [29.98] = 2.08, *p* = 0.04), while receiving fewer Alliance Bolstering (*t* [64] = 2.09, *p* = 0.04) and Empathetic Statements (*t* [64] = 2.27, *p* = 0.03) from their therapists, compared to participants with lower (<30%) change scores. Further independent t-tests showed that participants who reached clinical significance on the MPI – Pain interference scale received fewer Task Reinforcement (*t* [64] = −2.23, *p* = 0.03) and Alliance Bolstering (*t* [64] = −2.53, *p* = 0.01) statements from their therapists, while also receiving fewer coded behaviors in total (*t* [64] = −2.10, *p* = 0.04). Frequencies and relative proportions of all coded behaviors are presented in Table 4.

**Table 4:** Frequencies and proportions of coded behaviors.

Category	Total behaviors, n (%)	≥30% change^a^ behaviors, n (%)	<30% change^b^ behaviors, n (%)	Missing at post^c^ behaviors, n (%)
Participant behaviors	596 (100.00)	235 (39.43)	287 (48.15)	74 (12.42)
Facilitate understanding	76 (12.75)	22 (9.36)	49 (17.07)	5 (6.76)
Therapy process	51 (8.56)	16 (6.81)	28 (9.76)	7 (9.46)
Technical issues	118 (19.80)	43 (18.30)	62 (21.60)	13 (17.57)
Information request	0 (0.00)	0 (0)	0 (0)	0 (0)
Non-questions	351 (58.89)	154 (65.53)⁎	148 (51.57)⁎	49 (66.22)
Therapist behaviors	4788 (100.00)	1676 (35.00)	2343 (48.94)	769 (16.06)
Deadline flexibility	67 (1.40)	18 (1.07)	35 (1.49)	14 (1.82)
Task reinforcement	1297 (27.09)	455 (27.15)	645 (27.53)	197 (25.62)
Alliance bolstering	1816 (37.93)	685 (40.87)⁎	871 (37.18)⁎	260 (33.8)
Prompting	442 (9.23)	117 (6.98)	227 (9.69)	98 (12.74)
Psychoeducation	186 (3.90)	59 (3.52)	109 (4.65)	18 (2.34)
Self-disclosure	0 (0.00)	0 (0)	0 (0)	0 (0)
Self-efficacy shaping	9 (0.19)	3 (0.18)	3 (0.13)	3 (0.39)
Empathetic statements	230 (4.80)	92 (5.49)⁎	97 (4.14)⁎	41 (5.33)
Administrative statements	741 (15.48)	247 (14.74)	356 (15.19)	138 (17.95)

a Participants with ≥30% change from pre- to post-treatment on the Montgomery-Åsberg Depression Rating Scale (MADRS).

b Participants with <30% change from pre- to post-treatment on the Montgomery-Åsberg Depression Rating Scale (MADRS).

c Participants who did not fill out post-measurement questionnaires.

⁎ Significant differences between the groups (independent *t*-test, *p* < 0.05).

Correlational analyses showed several significant corrections (a < 0.01), both within and between participant and therapist behaviors. The more salient correlations are described below. See Table 5 for all correlations.

**Table 5:** Behavior intercorrelations.

	Participant behaviors^1^				Therapist behaviors^2^							
	1	2	3	4	5	6	7	8	9	10	11	12
Participant behaviors^a^												
1. Facilitate understanding	–											
2. Therapy process	0.35⁎⁎	–										
3. Technical issues	0.18	0.20⁎	–									
4. Non-questions	−0.10	−0.12	−0.08	–								
Therapist behaviors^b^												
5. Deadline flexibility	**0.13**	**0.27**⁎⁎	**0.00**	**0.07**	–							
6. Task reinforcement	**0.06**	**−0.05**	**0.05**	**−0.04**	−0.18	–						
7. Alliance bolstering	**0.39**⁎⁎	**0.27**⁎⁎	**0.33**⁎⁎	**0.30**⁎⁎	−0.02	−0.01	–					
8. Prompting	**−0.51**⁎⁎	**−0.32**⁎⁎	**−0.29**⁎⁎	**−0.18**	0.13	−0.25⁎	−0.66⁎⁎	–				
9. Psychoeducation	**0.45**⁎⁎	**0.26**⁎	**0.06**	**0.18**	0.20	−0.20⁎	0.44⁎⁎	−0.30⁎⁎	–			
10. Self-efficacy shaping	**0.22**⁎	**0.08**	**0.00**	**0.17**	0.19	−0.05	0.20	−0.20	0.17	–		
11. Empathetic statements	**0.35**⁎⁎	**0.19**	**0.06**	**0.41**⁎⁎	0.07	−0.35⁎⁎	0.37⁎⁎	−0.28⁎⁎	0.38⁎⁎	0.16	–	
12. Administrative statements	**−0.33**⁎⁎	**−0.05**	**−0.06**	**−0.20**	−0.12	−0.29⁎⁎	−0.61⁎⁎	0.30⁎⁎	−0.50⁎⁎	−0.17	−0.25⁎	–

Bold = correlations between participant and therapist behaviors.

a Proportions of participant behaviors relative to the total amount of participant behaviors coded for each participant.

b Proportions of therapist behaviors relative to the total amount of therapist behaviors coded for each participant.

⁎ *p* < 0.05.

⁎⁎ *p* < 0.01.

Correlations within participant behaviors showed that participant questions on treatment process (Therapy Process) were weakly correlated with questions about the treatment material (Facilitate Understanding; *r* = 0.35) and Technical Issues (*r* = 0.20). Non-questions showed no significant correlations with the other participant categories.

Within the therapist behaviors, Alliance bolstering showed moderate positive correlations with Empathetic Statements (*r* = 0.37) and Psychoeducation (*r* = 0.44). while correlating negatively with Prompting (*r* = −0.66) and Administrative Statements (*r* = −0.61), Finally, Psychoeducation showed a moderate negative correlation with Administrative Statements (*r* = −0.50). Of the observed behaviors, only Deadline Flexibility and Self-Efficacy Shaping lacked significant correlations with other therapist behaviors.

Correlations between participant and therapist behaviors showed participant Facilitate Understanding to be moderately correlated with both therapist Prompting (*r* = −0.51) and Psychoeducation (*r* = 0.45), while participant Non-questions correlated positively with therapist Empathetic Statements (*r* = 0.41). Only therapist Task Reinforcement lacked significant correlations with other participant behaviors.

Supplemental analysis of behavior frequency correlations showed significant positive correlations between all participant behaviors (*r* = 0.41–0.54). Similarly, all therapist behaviors were significantly and positively correlated with each other (*r* = 0.21–0.90) with the exception of a non-significant correlation between Empathetic Statements and Deadline Flexibility. Finally, analysis of correlations between participant and therapist behavior frequencies showed significant positive correlations between a majority of the behaviors (*r* = 0.28–0.74). Exceptions to this pattern was therapist Deadline Flexibility not correlating with participant questions on Technical Issues, therapist Prompting not correlating with any participant behavior except Non-questions, and Self-Efficacy Shaping only correlating with participant Facilitate Understanding and Non-questions. See Supplement A for all behavior frequency intercorrelations.

### 3.2 Adherence and outcome

No statistically significant correlations were observed between therapist or participant behavior proportions and change in the outcome measures, with the same being true for the frequency-based analysis. Several significant correlations were however observed between both therapist and participant behaviors and the two measures of adherence.

For participant behaviors, Facilitate Understanding (*r* = 0.43), Therapy Process (*r* = 0.20), Technical Issues (*r* = 0.38) and Non-questions (*r* = 0.33) were all significantly and positively correlated with treatment progress, and similar (but weaker) correlations were observed with treatment completion.

For therapist behaviors, five of the eight observed behaviors; Task Reinforcement (*r* = 0.32), Alliance Bolstering (*r* = 0.64), Psychoeducation (*r* = 0.43), Self-Efficacy Shaping (*r* = 0.24) and Empathetic Statements (*r* = 0.29), showed significant and positive correlations with treatment progress. Two of the behaviors; Prompting (*r* = −0.58) and Administrative Statements (*r* = −0.54) showed significant *negative* correlations with treatment progress. Finally, Deadline Flexibility showed no significant correlation with adherence. All correlations between coded behaviors and treatment adherence and outcome are presented in Supplement B.

## 4 Discussion

The purpose of the study was to characterize communications between participants and their therapists in guided and individually tailored iCBT for individuals with chronic pain and psychological distress. A secondary objective was to investigate if therapists and/or participant communication could relate to treatment adherence or outcome.

For participant behaviors, non-question-behaviors were the most frequently observed (59%), while amongst questions asked the most commonly observed were questions on technical problems (54%), followed by questions on the treatment material (34%), and process (23%). This can be compared to the previous study by Soucy (2019) where most participants questions were on the treatment material (47%) followed by the therapeutic process (23%), and technical issues (18%). Soucy et al. (2019) also observed many requests for additional information (12%), something that was not observed in the current sample (0%).

For therapists, the most frequently observed behaviors were Alliance Bolstering (38%) and Task Reinforcement (27%), indicating these behaviors to be prioritized by therapists in the current study. The third most commonly observed behavior was Administrative Statements (15%), potentially relating to the frequency of technical problems reported by participants. Some similar patterns can be observed compared to earlier studies, who also showed high frequencies of Task Reinforcement and low frequencies of Deadline Flexibility and Self-Disclosure (Paxling et al., 2012; Schneider et al., 2016). The dearth of Self-Efficacy Shaping in this study however (0%) contrast with Paxling et al. (2012) where it was frequently observed (34%). Infrequent Self-Efficacy Shaping (2%) was however also reported in the study by Schneider et al. (2016). The same authors also observed frequent Administrative Statements (16%), matching observations in the current study (15%).

In summary, more questions on Technical Issues amongst the participants and more Administrate Statements from therapists might indicate a high degree of IT-related difficulties in the current study. One reason for this could be that the treatment platform put a high priority of security, with negative consequences for user-friendliness. Requiring participants to use a code received by SMS for two-factor authentication, as well as the use of a PC rather than smartphones of tablets, might be one factor explain technical difficulties. Balancing security considerations with user convenience can be important to facilitate treatment adherence.

Correlational analyses within participant behaviors showed a higher proportion of Therapy Process-questions to correlate significantly with both questions on the treatment material (Facilitate Understanding. *r* = 0.35) and Technical Issues (*r* = 0.20), while Non-questions showed no correlations with other participant behaviors.

Within therapist behaviors, many correlations were observed, with the strongest being significant negative correlations between the proportions of Alliance Bolstering and both Prompting (*r* = −0.66) and Administrative Statements (*r* = −0.61). This could indicate that more frequent prompting and practical information can come at the expense of alliance building. This is however in contrast to previous studies which have shown no significant negative correlations between therapist behaviors. For example, both Paxling and Schneider (Paxling et al., 2012; Schneider et al., 2016) observed positive correlations between Alliance Bolstering and Prompting. A likely explanation for this discrepancy is the differing methodologies. While this study has explored relative proportions, earlier research focused on behavior frequencies. Indeed, our supplementary correlation analysis showed that using frequencies for this purpose yielded significant positive correlations between nearly all behaviors (see Supplement A for these results).

Correlational analyses between therapist and participant behaviors showed several significant correlations in the current sample. Participant questions about the treatment material was correlated with less therapist Prompting (*r* = −0.51) and more Psychoeducation (*r* = 0.45), which makes sense assuming that participants actively engaging with the material needs less prompting to do so. The category Non-questions also correlated with therapist Empathetic Statements (*r* = 0.41), potentially indicating that many non-question-statements were related to the participants mood or negative experiences. Only the therapist category Task Reinforcement did not correlate with any participant behavior. This might seem counterintuitive, since the purpose of reinforcement is to motivate behavior, but one explanation could be that task reinforcement disincentivizes further participant communication since it indicates to a participant that they are following the treatment correctly.

Analyses of adherence also showed several significant correlations. Participant questions all correlated positively with treatment progress, with questions on the treatment content showing the largest correlation (*r* = 0.43), and questions about the therapeutic process showing the smallest correlations with adherence (*r* = 0.20). This could indicate that participants who are focused on the content will adhere better to treatment as compared to those who are unsure about the treatment process. The weak correlations between questions regarding technical issues and adherence however is harder to interpret, but could possibly indicate that participants who are more engaged in treatment are also more invested in solving technical issues.

Therapist Prompting correlated negatively with treatment progress, which is expected since prompting is only necessary when a participant shows low adherence. Therapist Alliance Bolstering however, as well as Psychoeducation and participant questions on the treatment material (Facilitate Understanding), all correlated positively with treatment progress. This indicates that a style of correspondence that can improve adherence in iCBT consists of a therapist focused on alliance building and explaining psychological processes, and a participant who asks treatment-related questions.

No behavior proportions (or frequencies) amongst either therapists or participants correlated with treatment outcome (change scores on the MADRS or Pain Interference-scales). Coded behaviors relating more to adherence than outcome was expected, as the purpose of the therapist guidance is to assist participants and keep them engages with the treatment. However, a complete lack of outcome correlations is still in contrast to earlier studies showing significant negative correlations with outcome for some therapist behaviors (Paxling et al., 2012; Schneider et al., 2016), and the Paxling (2012) study in which Task Reinforcement related to reductions in worry. Assuming a relationship between adherence and outcome, an indirect relationship between behaviors and outcome might be expected.

One explanation for the lack of outcome correlations could be that the current coding schemes are missing an important aspect of the correspondence. For example, many participant statements were not questions, and there might be other factors in participant non-question-correspondence that would relate more to outcome. A different explanation could be that relationships to outcome would be stronger with behaviors not commonly observed in this study. For example, therapists in the current study used very little Self-Disclosure and few Empathetic Statements, which might be important to include in communication. While carefully using self-disclosure to bolster alliance was part of the basic training of all therapist in this study, therapists received no specific instructions to do so, or how much. It is possible that therapists worried about negative responses might have hesitated in using self-disclosure. Of course, the reverse argument is also possible. Maybe specific behaviors are less important for outcome than overall tone, density of contact, therapeutic alliance, or engagement.

Finally, when looking at clinically significant change, a clear pattern emerged where participants who attained ≥30% improvement exhibited fewer coded behaviors in total (with the exception on non-questions), and also received fewer behaviors from their therapists. This indicates that therapists, in line with their training, adapt responses to participant needs, with more attempts at communication and alliance building aimed at participants who experience difficulties in the treatment.

### 4.1 Limitations

This study has several limitations. First, correlational analyses cannot reveal causal relationships, and no conclusions can be drawn about what questions result in which answers, or what therapist behaviors prompt which questions. It is also impossible to tell whether a certain type of communication can promote adherence, or if the adherence promotes this type of communication. Likely the actual relationships are bidirectional, requiring more complex analyses to elucidate these issues further.

Second, coding text is inherently subjective, and though differences between categories are clear in many cases, in others a large degree of subjective judgement is needed. For example, asking a participant “how is it going with the week's exercises?” might be construed as Alliance Bolstering, but depending on context might well be an attempt at Prompting a participant with low adherence to continue treatment. In some sense most therapist statements could be viewed as a type of prompting since their main purpose could be viewed as affecting a treatment-related response from the participants. In general, differentiation between Alliance Bolstering, Prompting, and Task Reinforcement were especially prone to difficulties. Potentially, further refinement of these coding schemes could yield more consistent results.

Third, this study used relative proportions of behaviors rather than frequencies, which both limit comparisons to previous research, and introduces several methodological limitations. Relative values means that an increase in one behavior proportion necessitates a decrease in the others, which could hide or inflate correlations. While previous studies have instead used behaviors frequencies as the basis for correlational analyses, this presents other issues. As the number of modules completed (i.e., adherence) increases, the number of messages sent, and thus behaviors exhibited, will naturally grow as well, making behavior frequencies themselves a proxy for adherence. Additionally, when analyzing correlations within therapist or participant behaviors, all relationships will trend to the positive, since more frequent communication or longer messages will always imply a larger number of behaviors for coding. Finally, more frequent questions from participants will naturally result in more frequent answers from therapists, and so on. Especially when looking at relationships between participant and therapist behaviors, this might inflate the number of significant correlations and obscure potentially negative relationships. Using proportions does not solve this problem entirely however, since a larger message volume will still produce more stable proportional estimates.

### 4.2 Strengths

Several strengths of this study also bear mentioning. First, we recruited participants from specialist care with high degree of comorbidity and pain-related dysfunction, which improves ecological validity and adds to the research on a less well studied population. To our knowledge, this is also the first study investigating text correspondence in participants with chronic pain, which is especially important given the issues with low adherence in this population. This study might give further insights into the relationship between adherence and correspondence in iCBT for these patients.

Secondly, coding was based on previously identified themes from previous research, partially extending and adapting it to a new sample. Even though the themes were adapted to the sample, this enables some comparisons with previous research and could help with further refinements of these coding schemes. Coding was also conducted by two coders with frequent discussions promoting further adaptations of the coding schemes.

Finally, to our knowledge this is the first study describing not just therapist or participant communication, but also relationships between them. Indeed, it has been observed that most previous studies focus on therapist behaviors, with very limited research on the interactions between therapists and participants in iCBT (González-Robles et al., 2024). This study could potentially add to existing research by inspiring future studies on casual relationships between therapist and participant behaviors. Such research could be useful for clinicians using internet-based methods, as well as providing clear guidelines for educators specializing in teaching or supervising internet-based treatments.

### 4.3 Summary and future directions

With text-correspondence being the primary task of any therapist working with internet-based treatments, optimizing this correspondence could potentially reduce treatment costs and attrition, while improving outcomes. As current research on this topic is very limited, future clinical researchers should consider analyzing not only therapist behaviors, but also participant correspondence. While the internet-based format vastly simplifies data collection, scaling this type of research presents many practical issues. Future studies could potentially use tools such as large language models (LLMs) for less resource intense coding, enabling larger samples and more advanced statistical analyses. Additionally, recognizing that many participant messages will contain things other than questions, a broader qualitative investigation of participant behaviors in internet-based treatments seem warranted. Finally, there are other process variables that warrant consideration. As therapists commonly focus on empathy and alliance building, investigating the therapeutic alliance more specifically, and its relationship to both therapist and participant behaviors, might guide future clinicians in adapting their communication to optimize alliance. Finally, other potential mediators such as psychological flexibility and catastrophizing might also merit further investigation in the context of communication. Investigating what types of correspondence might enhance these factors could be especially relevant in studies of chronic pain, since both have been shown to mediate treatment effects on disability (Murillo et al., 2022).

In summary, this study indicates that distinct therapist and participant behaviors can be identified in iCBT, though frequencies of these behaviors seem to differ between studies. The current study also found that many of these behaviors correlate. Mainly, the pattern of correlations revealed that participants treatment-related questions correlated with more psychoeducation and less prompting from therapists. While several of the identified behaviors correlated with treatment adherence, like therapist alliance building or participant questions on the treatment material, any direct relationships with outcome were less clear. More research is needed to further investigate whether some specific behaviors or styles of communication might affect treatment outcomes directly.

## Ethics approval and consent to participate

The study was approved by the Uppsala Regional Ethics Review Board (2016/107) and the study was conducted in accordance with relevant guidelines and regulations. Written informed consent was collected from all participants.

## Funding

This study was funded in collaboration between 10.13039/501100007051Uppsala University and 10.13039/501100002706AFA Insurance.

## Declaration of competing interest

The authors declare that they have no competing interests.

## Data Availability

The dataset analyzed during the current study are available from the corresponding author on reasonable request.

## Appendix A Supplementary data

Supplementary material 1 Behavior frequency intercorrelations.

Supplementary material 2 Behavior correlations with adherence and outcome.
