# Supplementary material for: Therapist-patient correspondence in internet-based CBT for chronic pain: Associations with outcome and adherence
Source: Internet Interv. 2026 Jun 4;45:100958. doi: 10.1016/j.invent.2026.100958 (PMC13266229; doi:10.1016/j.invent.2026.100958)
Supplement: Supplementary material 1 — Behavior frequency intercorrelations. [file mmc1.docx]

| **Supplement A.** Behavior frequency intercorrelations | | | | | | | | | | | | |
| --- | --- | --- | --- | --- | --- | --- | --- | --- | --- | --- | --- | --- |
|  | **Participant behaviors^1^** | | | | **Therapist behaviors^2^** | | | | | | | |
|  | **1** | **2** | **3** | **4** | **5** | **6** | **7** | **8** | **9** | **10** | **11** | **12** |
| **Participant behaviors^1^** |  |  |  |  |  |  |  |  |  |  |  |  |
| 1. Facilitate Understanding | **-** |  |  |  |  |  |  |  |  |  |  |  |
| 2. Therapy Process | .45^**^ | - |  |  |  |  |  |  |  |  |  |  |
| 3. Technical Issues | .41^**^ | .43^**^ | - |  |  |  |  |  |  |  |  |  |
| 4. Non-Questions | .37^**^ | .37^**^ | .54^**^ | - |  |  |  |  |  |  |  |  |
| **Therapist behaviors^2^** |  | | | | | | | | | | | |
| 5. Deadline Flexibility | **.27^**^** | **.29^**^** | **.14** | **.30^**^** | - |  |  |  |  |  |  |  |
| 6. Task Reinforcement | **.48^**^** | **.37^**^** | **.51^**^** | **.64^**^** | .26^**^ | - |  |  |  |  |  |  |
| 7. Alliance Bolstering | **.53^**^** | **.44^**^** | **.59^**^** | **.74^**^** | .31^**^ | .90^**^ | - |  |  |  |  |  |
| 8. Prompting | **-.04** | **.07** | **.17** | **.28^**^** | .44^**^ | .35^**^ | .38^**^ | - |  |  |  |  |
| 9. Psychoeducation | **.57^**^** | **.38^**^** | **.32^**^** | **.52^**^** | .29^**^ | .54^**^ | .70^**^ | .28^**^ | - |  |  |  |
| 10. Self-Efficacy Shaping | **.30^**^** | **.16** | **.20** | **.36^**^** | .32^**^ | .26^*^ | .31^**^ | 0.15 | .27^**^ | - |  |  |
| 11. Empathetic Statements | **.52^**^** | **.33^**^** | **.34^**^** | **.64^**^** | .19 | .56^**^ | .71^**^ | .21^*^ | .61^**^ | .30^**^ | - |  |
| 12. Administrative  Statements | **.45^**^** | **.47^**^** | **.60^**^** | **.70^**^** | .27^**^ | .82^**^ | .85^**^ | .38^**^ | .52^**^ | .28^**^ | .57^**^ | - |
| * = p <.0.05, ** = p <.0.01  1. Frequency of participant behaviors  2. Frequency of therapist behaviors  **Bold** = correlations between participant and therapist behaviors | | | | | | | | | | | | |
