# Supplementary material for: Therapist-patient correspondence in internet-based CBT for chronic pain: Associations with outcome and adherence
Source: Internet Interv. 2026 Jun 4;45:100958. doi: 10.1016/j.invent.2026.100958 (PMC13266229; doi:10.1016/j.invent.2026.100958)
Supplement: Supplementary material 2 — Behavior correlations with adherence and outcome. [file mmc2.docx]

| **Appendix B.** Behavior correlations with adherence and outcome | | | | |  |
| --- | --- | --- | --- | --- | --- |
|  | **Treatment adherence** | | **Outcome change scores** | |  |
| **Participant behaviors^1^** | **Treatment progress (%)^2^** | **Completed Treatment^3^** | **MADRS^4^** | **Pain interference^4^** |  |
| Facilitate Understanding | .43^**^ | .31^**^ | .02 | .2 |  |
| Therapy Process | .20^*^ | .13 | .16 | -.11 |  |
| Technical Issues | .38^**^ | .27^**^ | -0.03 | 0.09 |  |
| Non-questions | .33^**^ | .31^**^ | -0.20 | -0.15 |  |
| **Therapist behaviors^1^** |  | | | |  |
| Deadline Flexibility | .06 | -.06 | 0.07 | -0.04 |  |
| Task Reinforcement | .32^**^ | .32^**^ | -0.05 | 0.02 |  |
| Alliance Bolstering | .64^**^ | .45^**^ | -0.08 | -0.14 |  |
| Prompting | -.58^**^ | -.50^**^ | 0.05 | 0.06 |  |
| Psychoeducation | .43^**^ | .28^**^ | 0.02 | -0.11 |  |
| Self-Efficacy Shaping | .24^*^ | .22^*^ | -0.08 | 0.19 |  |
| Empathetic Statements | .29^**^ | .15 | 0.07 | 0.11 |  |
| Administrative Statements | -.54^**^ | -.43^**^ | -0.04 | 0.00 |  |
| *MADRS*. Montgomery-Åsberg Depression Rating Scale; *Pain interference*. Multidimensional Pain Inventory – Pain Interference subscale  1. Proportions of coded behaviors relative to the total amount of behaviors coded for each participant/therapist  2. Proportion of assigned modules completed (%)  3. Completed ≥ 75% of assigned modules (1/0)  4. Residualized change from pre- to post-treatment  * = p < .05. ** = p < .01 | | | | | |
